# On-chip integrated graphene aptasensor with portable readout for fast and label-free COVID-19 detection in virus transport medium†

**DOI:** 10.1039/d2sd00076h

**Published:** 2022-06-13

**Authors:** Lizhou Xu, Sami Ramadan, Bruno Gil Rosa, Yuanzhou Zhang, Tianyi Yin, Elias Torres, Olena Shaforost, Apostolos Panagiotopoulos, Bing Li, Gwilherm Kerherve, Dong Kuk Kim, Cecilia Mattevi, Long R. Jiao, Peter K. Petrov, Norbert Klein

**Affiliations:** a Department of Materials, Imperial College London London SW7 2AZ UK l.xu@imperial.ac.uk n.klein@imperial.ac.uk; b ZJU-Hangzhou Global Scientific and Technological Innovation Center, Zhejiang University Hangzhou 311200 China; c Hamlyn Centre, Imperial College London London SW7 2AZ UK; d Graphenea Semiconductor Paseo Mikeletegi 83 San Sebastián 20009 Spain; e Department of Brain Sciences, Imperial College London London W12 0BZ UK; f Care Research & Technology Centre, UK Dementia Research Institute W12 0BZ UK; g Institute for Materials Discovery, University College London Roberts Building London WC1E 7JE UK; h Department of Hepatobiliary Surgery, Division of Surgery & Cancer, Imperial College London Hammersmith Hospital Campus, Du Cane Road London W12 0NN UK

## Abstract

Graphene field-effect transistor (GFET) biosensors exhibit high sensitivity due to a large surface-to-volume ratio and the high sensitivity of the Fermi level to the presence of charged biomolecules near the surface. For most reported GFET biosensors, bulky external reference electrodes are used which prevent their full-scale chip integration and contribute to higher costs per test. In this study, GFET arrays with on-chip integrated liquid electrodes were employed for COVID-19 detection and functionalized with either antibody or aptamer to selectively bind the spike proteins of SARS-CoV-2. In the case of the aptamer-functionalized GFET (aptasensor, Apt-GFET), the limit-of-detection (LOD) achieved was about 10^3^ particles per mL for virus-like particles (VLPs) in clinical transport medium, outperforming the Ab-GFET biosensor counterpart. In addition, the aptasensor achieved a LOD of 160 aM for COVID-19 neutralizing antibodies in serum. The sensors were found to be highly selective, fast (sample-to-result within minutes), and stable (low device-to-device signal variation; relative standard deviations below 0.5%). A home-built portable readout electronic unit was employed for simultaneous real-time measurements of 12 GFETs per chip. Our successful demonstration of a portable GFET biosensing platform has high potential for infectious disease detection and other health-care applications.

## Abstract 2

On-chip integrated graphene field-effect transistor (GFET)-based aptasensor was developed with portable readouts for sensitive and specific virus detection.

## Graphical abstract

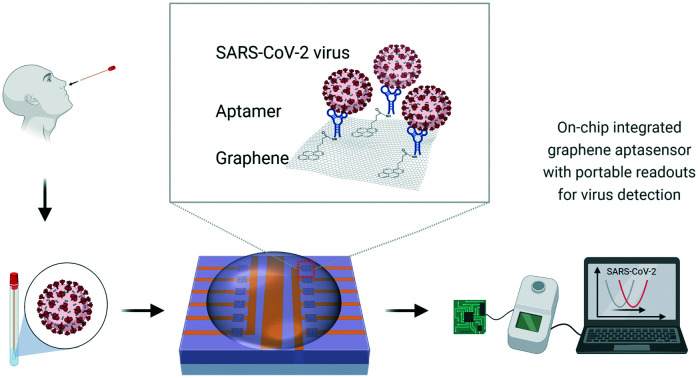

## 1 Introduction

Graphene exhibits remarkable properties, including ultrahigh carrier mobility and thermal conductivity as well as attractive optical properties.^1–3^ This has led to the realization of various novel devices based on graphene in a wide range of fields, including optics, MEMS, nanoelectronics and sensors.^4^ The graphene field-effect transistor (GFET) is one of the main sensing platforms suitable for bio-detection and bio-analysis.^5^ The adaptation of GFET technology for biosensing could lead to simple and sensitive diagnostic devices. A GFET exhibits ambipolar charge transport behaviour in which electrons and holes can be modulated using an external electric field.^6,7^ Its two-dimensional structure consisting of a graphene monolayer one atom thick facilitates strong responses to charged molecules present on the surface.^8^ More importantly, most biomolecules are naturally charged and so GFET sensors allow intrinsically label-free detection.^9–13^ The readout signal of a GFET is electrical and therefore compatible with electrical measurements based on standard electronic circuits, state-of-the-art data recording, data storage, screen display, and wireless connectivity. Furthermore, monolayer graphene is biofriendly, allowing biofunctionalization and bioconjugation with a variety of biological reagents and playing an increasingly important role in biosensing.^14^ Graphene growth *via* chemical vapour deposition and subsequent transfer onto large-area silicon wafers has steadily advanced, allowing the large scale production of sensor devices using CMOS-compatible wafer-scalable processes.^15^ Along with scalable semiconductor manufacturing techniques that enable a dramatic miniaturization of chip footprints without sacrificing performance, the implementation of disposable sensor chips at low cost with multi-target detection capability is now realistic. Since the power consumption per GFET is of the order of microwatts, portable and battery-powered sensor readout devices^14^ for multiple chips should enable use at points-of-care and checkpoints or in the home.

GFET biosensors have been demonstrated for the detection of SARS-CoV-2 viruses,^13^ neutralizing antibodies,^13,16,17^ and other human-infectious viruses such as Zika,^18^ avian influenza,^19,20^ Ebola,^21^ and the human immunodeficiency virus (HIV).^22^ They have also been used for the detection of a wide range of biomolecules, including proteins,^23,24^ DNA,^25–27^ small molecules,^28–30^ other disease biomarkers.^31–33^ Nevertheless, there are practical limitations that prevent their broad implementation in real applications.^6,34^ For example, GFETs often employ Ag/AgCl reference electrodes which are bulky and difficult to integrate for detection in small sample volumes of less than a few microlitres, where the latter are sufficient for detection by the GFET alone. Moreover, external reference electrodes add complexity and cost.

**Fig. 1:**
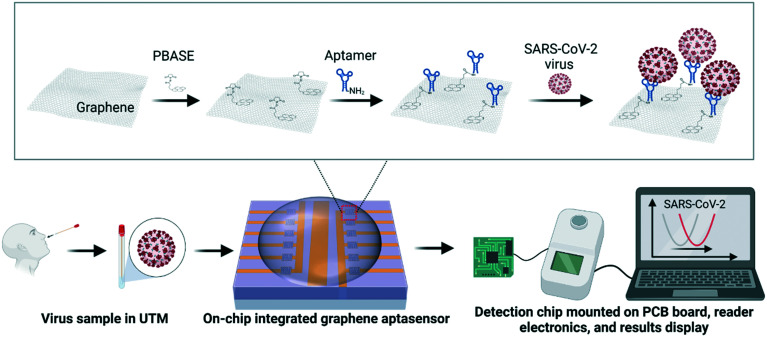
Schematics of the SARS-CoV-2 virus detection using an on-chip integrated GFET aptasensor with portable electronics and the software presenting real-time detection results on a laptop. The zoomed-in area in the middle shows the schematics of the functionalization of the aptamer. The chip layout allows the different functionalization of two sections on a chip comprising 6 GFETs each.

Here we report on stable and highly sensitive GFET biosensor arrays with portable read-in/out electronics. This tunable platform technology allows the bio-decoration of single-layer graphene with aptamers, antibodies, and recombinant proteins for high-performance detection. We not only show the excellent detection performance of on-chip GFET biosensors for targets dispersed in buffer solutions, but also for virus-like-particle (VLP) samples dispersed in universal transport medium (UTM) for SARS-CoV-2 swab sample storage, as well as the detection of COVID-19 antibodies in serum conditions. Fig. 1 shows the schematics of the SARS-CoV-2 VLP detection using an on-chip integrated GFET aptasensor with portable electronics and the software presenting real-time detection results on a laptop. A specific aptamer sequence was used for the bio-recognition and capturing of spike proteins of target SARS-CoV-2 viruses. A schematic of the functionalization process for aptamers is shown in the zoomed-in area of Fig. 1.

## 2 Experimental section

### 2.1 Reagents

The spike proteins of SARS-CoV-2 were purchased from Sino Biological, Germany (40591-V08H). The SARS-CoV-2 virus-like-particles (VLPs, VLP-050YF) with structural proteins (spike, membrane, and envelope) assembled were purchased from Creative Biolabs, USA. The sequence of the spike protein aptamer is −5′ GTC TTG CGG GGC GGC GGG TTG AGA GGA 3′-.^35^ The 3′ end was conjugated with the functional group –NH_2_ for conjugation with the *N*-hydroxysuccinimide (NHS) group on the PBASE linker molecule. The aptamer sequences were synthesized by Integrated DNA Technologies, Belgium. The rabbit anti-SARS-CoV-2 spike protein S1 monoclonal antibody was purchased from Sino Biological, Germany (40150-R007). Universal transport medium (UTM) medium for virus specimen collection and transportation was obtained from the Imperial Testing Centre and supplied as Sigma Virocult (large) Media (MW950T) by MWE, UK.

### 2.2 Fabrication of on-chip integrated graphene sensor array (GFET sensor chip)

**Fig. 2:**
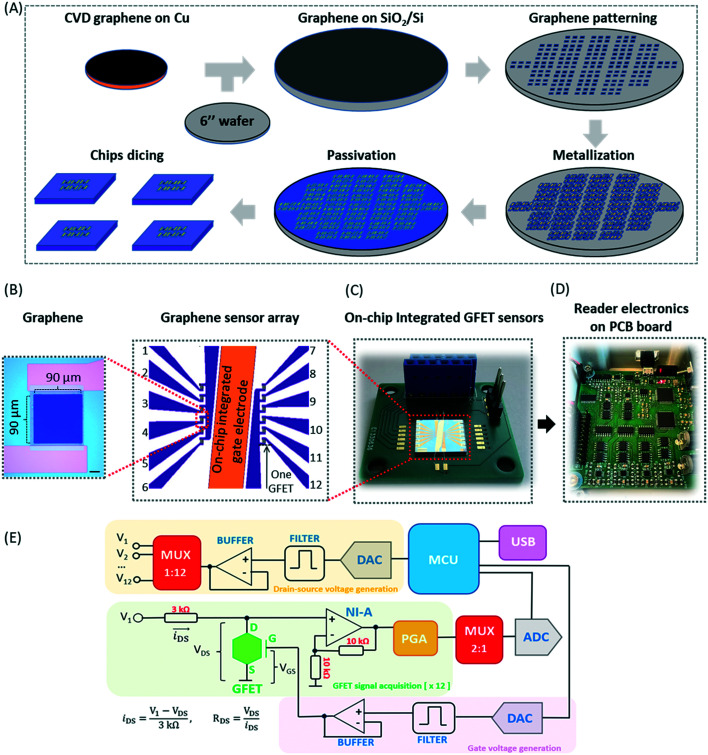
Illustration of the graphene chip, GFET array, and PCB board-based portable electronics: (A) process steps of wafer scale GFET fabrication; (B) chip design of the on-chip GFET array with an on-chip integrated gate electrode, where on-chip GFET sensors are designed with 6 devices each on the left and right sides and the left-hand enlarged image shows the graphene deposited between source and drain electrodes with a scale bar of 20 μm; (C) image of the integrated on-chip sensor; (D) image of the PCB board with reader electronic components; (E) simplified electronic schematic highlighting the main functional blocks and components of the reader electronics on the PCB board (MCU – microcontroller, DAC – digital-to-analogue converter, ADC – analogue-to-digital converter, MUX – multiplexer, USB – universal serial bus, PGA – programmable gain amplifier, NI-A – non-inverting amplifier), with the mathematical formulae involved in the estimation of the drain–source current and resistance exhibited on the bottom left corner.

The graphene used in the on-chip GFETs is high-quality monolayer CVD graphene and its fabrication and manufacturing processes are highly compatible with conventional CMOS technology. The graphene sensor array was designed with on-chip liquid gold (Au) gate electrodes (integrated GFETs) and manufactured on 100 mm and 150 mm wafer in the Graphenea foundry (https://www.graphenea.com/). Fig. 2A shows the wafer scale manufacturing process. The GFETs with integrated Au gate electrodes are manufactured with a combination of CVD graphene growth on Cu, graphene transfer onto Si/SiO_2_ substrate, graphene patterning using a combination of optical lithography and O_2_ plasma etching, metallization, passivation, and chip dicing. An aluminum dioxide film was used to passivate the devices due to its excellent chemical stability.^36^ The carrier mobilities of individual GFETs, as determined from the measured IV-characteristics employing Si/SiO_2_ bottom gates, are around 1700 cm^2^ V^−1^ s^−1^ with standard deviations *σ* = 280 cm^2^ V^−1^ s^−1^ determined for 10 batches of devices (batch-to-batch variation) and *σ* = 340 cm^2^ V^−1^ s^−1^ for device-to-device variation within one wafer. The average yield for each wafer is >95% according to quality control measurements on a larger number (*n* = 310) of chips.

The GFET sensor chip with integrated on-chip liquid electrode is shown in Fig. 2B. The GFET sensor chip (10 mm × 10 mm) consists of a central gold (Au) gate electrode with in-plane position (rather than an external bulky gate electrode) and 12 individual GFET devices with a graphene channel area of 100 μm × 100 μm and a window opening of 90 μm × 90 μm deposited between the source and drain electrodes (see optical image inset in Fig. 2B). The 12 individual graphene devices are separated into two distinct sections by a central on-chip Au gate electrode allowing liquid gating without the need for a bulky external gate electrode. Since Au is a polarized electrode, the application of an electric field at the gate electrode will lead to the formation of a double layer not only at the graphene sensor surface but also at the Au electrode. The total capacitance is series capacitance given by 
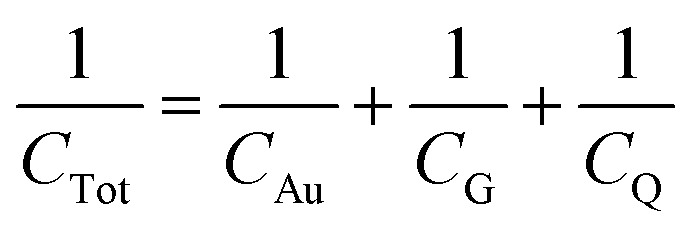
, where *C*_Au_ and *C*_G_ are the electrical double layer capacitances at Au, and G respectively and *C*_Q_ is the quantum capacitance of graphene. Therefore, to minimize the impact of the electrical double-layer capacitance at the gate electrode, the area of the Au gate electrode is designed to be much larger than that of the graphene sensor (*A*_Au_ > 1000 × *A*_G_).^37,38^ The Debye length in 1000 diluted PBS is 23 nm. The electrical double layer at graphene is calculated to be 
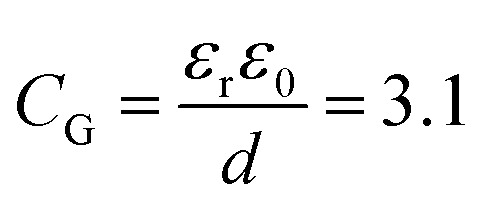
 μF cm^2^. The experimental value of quantum capacitance is reported to be between 2–11 μF cm^2^ which depends on the gate potential and charged impurities.^39,40^ Therefore, the total capacitance should take into account both the quantum capacitance and graphene's electrical double-layer capacitance. Incorporating internal controls within one detection chip is necessary in order to distinguish between target and interfering molecules or background noise, which enables chip calibration. In our design, the six GFETs in each functional area usually operate as independent internal controls during the same chip measurements and enable the statistical analysis and monitoring of device-to-device variation, facilitating an error-reducing detection process.

### 2.3 Development of the portable readout system

A portable readout system was designed to measure the electrical response of GFETs in a fast and user-friendly way. This system consists of twelve independent channels for drain-source voltage acquisition and stimulation. Twelve drain electrodes are connected to a GFET chip, whereas two source electrodes are connected from the chip to the electronic ground of the measurement device, thereby closing the path for electrical current circulation inside each individual channel (*i*_DS_). A single floating gate connection (electrode) can also be established between the GFET chip and the measurement device (Fig. 2C and D). Simultaneous voltage signals (*V*_1_ to *V*_12_) are generated through a digital-to-analogue converter (DAC) operated by a central microcontroller (MCU) that releases the digital samples in a timely manner, followed by low-pass filtering and the signal buffering of the analogue equivalent level carried by each digital sample, before being fed into a multiplexer (MUX) that selects the channel to be stimulated inside the GFET chip from among the 12 available channels. This allows the production of voltage signals for drain–source stimulation in the DC and AC regimes by the user which are selected with an amplitude ranging from −8 V to +8 V (steps of 250 μV) and a bandwidth of 15 kHz (Fig. 2E, drain–source voltage generation). The drain–source current is then measured inside a typical voltage-divider topology created between the drain and source (ground) terminals of a single GFET channel. The voltage signal (*V*_DS_) is amplified by a non-inverting amplifier (NI-A) with a gain set to 2 V/V, followed by a programmable gain amplifier (PGA) with user-selectable gain (1, 10, 100 and 1000 V/V) to further discriminate between the drain–source voltages detected. A 2 : 1 signal multiplexer then routes the detected voltages from the 12 acquisition channels (Fig. 2E, GFET signal acquisition [×12]) to interface an 8-channel analogue-to-digital converter (ADC) with 16-bit resolution. Six of the ADC channels sample alternatively the 12 acquisition channels in groups of 6, whereas the remaining two channels track the imposing voltage signal involved in the drain–source stimulation and the floating gate potential (*V*_GS_), where the latter is generated by an identical chain of electronic modules (Fig. 2E, gate voltage generation). Both signals are digitized at a rate of 30 kSPS inside the ADC and stored inside the MCU before transfer to the computer through a universal serial bus (USB) at speeds of 1 Mbps. Finally, the entire system is powered by a ±9 V power supply (current consumption of 60 mA) and controlled by a graphical user interface (GUI) developed in Matlab (Mathworks Inc., USA), where different measurement modes can be performed, including of resistance, Dirac's point, and time for a single channel or all channels simultaneously (Fig. S1†).

### 2.4 Biofunctionalization of GFETs and characterization of on-chip integrated GFET sensors

Biofunctionalization usually includes an incubation step to deposit a single molecular layer of linker molecule 1-pyrenebutanoic acid succinimidyl ester (PBASE), and a subsequent deposition of biological reagents for target capture and measurement. First, the GFET devices were incubated with PBASE (10 mM in dimethylformamide (DMF) (Sigma-Aldrich)) for 2 h at room temperature and then gently rinsed in DMF to remove excess PBASE from the surface before being dried with N_2_. Samples were then conjugated with either aptamer against the spike protein of SARS-CoV-2 (scheme inset in Fig. 1) or rabbit anti-SARS-CoV-2 spike protein S1 monoclonal antibody. Droplets of incubation solution 20 μL in volume were added to the surface of the chip and left overnight in a humidified environment at 4 °C. The chip was then sequentially rinsed in 1× PBS and de-ionized (DI) water and dried with N_2_. The chip was blocked using 3% bovine serum albumin (BSA) in 1× PBS for 1 h and then rinsed with PBS and DI water and dried with N_2_ prior to the electrical measurements.

Results for the characterization of on-chip GFET sensors, including atomic force microscopy (AFM), and Raman and X-ray photoelectron spectroscopy (XPS), are provided in the ESI.†

### 2.5 Analytical performance assessment of the on-chip integrated GFET sensors for spike protein detection in PBS buffer

Immediately following the incubation and cleaning of the GFET sensor chip, electrical measurements were performed in 0.001× PBS solution (d1000 PBS). Source–drain voltage was fixed at 20 mV and the electrolyte gate was swept from 0.2 to 0.9 V at a rate of 30 mV s^−1^, rendering source–drain currents in the order of 10 s of microampere (μA) in d1000 PBS and UTM. Samples were tested using the developed on-chip GFET sensors and the transfer curves were recorded. For the investigation of selectivity, MERS-CoV spike/S1 protein (40069-V08B1, Sino Biological, Germany), human coronavirus (HCoV-229E) spike/S1 protein (40601-V08H, Sino Biological, Germany), and BSA (Sigma-Aldrich, UK) were used. For time-resolved measurements, spike protein samples at various concentrations were added to the functionalized sensor surface one by one, followed by a PBS wash-up step in between each sample.

### 2.6 Detection of SARS-CoV-2 virus-like-particles (VLPs) in real biofluids (universal transport medium, UTM)

The VLP stock (0.13 mg mL^−1^, about 10^9^ particles per mL, in supplier's buffer of 20 mM Tris-Cl, pH 7.9, 100 mM NaCl and 0.5 mM EDTA) were mixed with UTM at 1 : 1 (v : v) ratio to mimic the COVID-19 positive swab samples. Then the non-infectious VLP in UTM stock sample was diluted to 10^3^–10^7^ particles per mL using d1000 PBS before performing the measurement protocol using the on-chip biosensor. The electrical signal correlates with the concentration of VLPs.

### 2.7 Statistical analyses

For all experiments, data are presented as mean ± SD, where n denotes the number of replications. The statistical significance of the data was assessed using the Student's *t*-test and is designated with asterisks (p* < 0.05, p** < 0.01, and p*** < 0.001).

## 3 Results and discussion

### 3.1 Characterization and quality assessment of biofunctionalized on-chip integrated graphene sensor array (GFET sensor chip)

**Fig. 3:**
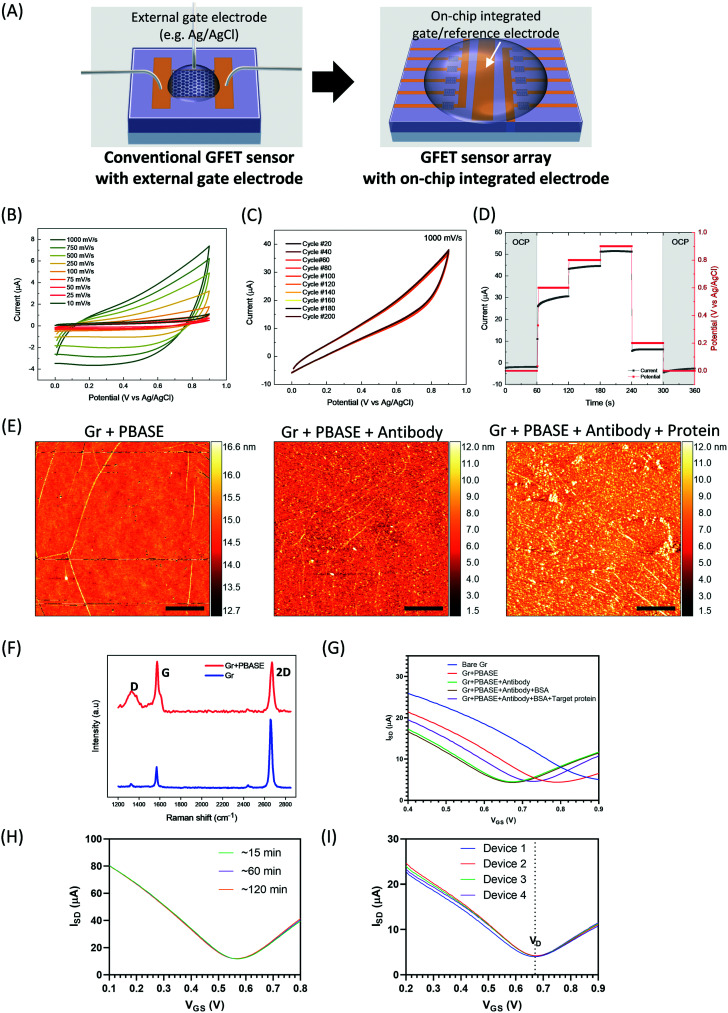
Characterization and quality assessment of the on-chip GFET sensing platform: (A) the design of on-chip integrated sensor array outperforms the conventional GFET single sensor which uses an external bulky gate electrode (*e.g.* Ag/AgCl) and has minimal detection throughput; (B–D) electrochemistry of the device in a three-electrode set-up with an Ag/AgCl reference electrode and carbon foil counter electrode in 0.001 M PBS solution of pH 7.4; (B) the cyclic voltammogram of the device contacted at the gold/aluminum oxide junction in a scan rate range of 1000–1000 mV s^−1^; (C) cyclic voltammogram of the device contacted on gold showing a stability test done at a 1000 mV s^−1^ over 200 cycles; (D) the chronoamperometry experiment contacted on gold showing the current measured in response to the open circuit potential and applied potential; (E) AFM images showing the successful functionalization/binding of the PBASE, the antibody, and the target protein, with scale bar: 1 μm; (F) the Raman spectroscopy before and after PBASE functionalization on graphene surface; (G) *I*–*V* curves showing changes in the Dirac point after each surface functionalization step, where electrical measurement (*I*–*V* curves) confirmed the successful functionalization/binding of each molecule on the graphene surface (330 fM of spike proteins); (H) monitoring of *I*–*V* curves over time on the same device (33 pM of spike proteins). *V*_sd_ = 20 mV, indicating good stability of the data produced by each device over time; (I) *I*–*V* curves indicating good data repeatability and the signal stability of multiple sensing devices (*n* = 4) at 330 pM of spike proteins in d1000 PBS.

In comparison to the conventional GFET single sensor which needs a bulky external gate electrode (such as Ag/AgCl), an on-chip GFET sensor is advantageous not only because of the use of an integrated on-chip gate/reference electrode in the centre area which avoids external bulk electrodes and a complicated set-up. Embedding an internal control on the chip can help in distinguishing between signals caused by target species and those caused by the buffer background or interfering species (Fig. 3A). Hence, interfering signals can be normalized and internally calibrated by measuring the difference in response on the same chip between the sensor (for example, six GFETs in parallel on the left side which were functionalized with antibody and used for target detection in a solution) and control (six GFETs in parallel on the right side which were also functionalized with antibody in the same way but used for detection of the background signal in the same solution) devices, which should lead to improved sensing accuracy and reliability.

In order to examine the stability of the on-chip integrated GFET sensor, we performed a series of electrochemical and electrical measurements. To unequivocally demonstrate the device's stability in PBS solution, we first performed a series of cyclic voltammograms and chronoamperometry measurements contacting the device across all components. The cell's operating potential window was monitored in the 0–0.9 V range and an operating time in the 0.5–180 s range so as to reflect the required sensing conditions. The junction between gold and aluminum oxide is particularly important for investigation, since this is where the potential is applied and the current is measured. The device shows high stability as indicated by the metallic behaviour and electrostatic steady state reactance, with no defined redox peaks, fast charge transfer reactions and negligible drift over 200 cycles (Fig. 3B and C). Negligible drift refers to a current drift due to an irreversible unexpected reaction. Furthermore, chronoamperometry measurements suggest that there is a linear metallic response with no energy demand or release in any of the reactions occurring at the electrode/electrolyte interface (Fig. 3D). The stability and non-reactivity of the Au gate electrode with buffer solution, confirmed by series of electrochemical and electrical measurements, supports the potential for this technology to replace the bulky Ag/AgCl reference electrode that limits the miniaturization of GFET sensor technology.

To demonstrate biocompatibility, we first biofunctionalized the on-chip GFET with PBASE and spike protein-specific antibodies on the single graphene layer, and then utilized this on-chip GFET sensor in an electrolyte-gated way for the sensing of the spike protein of SARS-CoV-2. The introduction of charged spike proteins onto the GFETs leads to a change in the doping state of the graphene, which changes the position of the Dirac voltage (the gate voltage which is needed to compensate for the internal doping of graphene *via* the ambipolar field-effect). The Dirac voltage correlates with the target protein concentration. Various methods were used for characterization. AFM images show the successful functionalization/binding of PBASE, antibody, and target protein (Fig. 3E). The graphene surface before and after PBASE functionalization was studied using Raman spectroscopy (Fig. 3F). The as-transferred graphene showed high-quality single-layer graphene with a low density of defects. The PBASE-coated graphene exhibits a more pronounced disordered D peak at around 1350 cm^−1^ due to the resonance of sp^3^ bonding or interaction between the localized vibrational modes of PBASE and the extended phonon modes of graphene.^41^ The measured *I*–*V* transfer curves (Fig. 3G) show changes in the Dirac voltage after each step of the surface functionalization. The negatively charged spike proteins (Fig. S2†) induced p-doping in graphene, causing a significant positive shift in Dirac voltage after proteins binding. Optical and electric characterizations confirmed that the functionalization/binding of each molecule on the graphene surface was successful.

To further quantify stability and repeatability, electrical measurements were performed. We first measured the stability of the devices in PBS by recording the changes in transfer curves after repeating the measurements several times. We observed that devices sometimes showed some drift, which can range from a few mV for some chips up to 200 mV in others. However, the drift usually vanishes after the first five measurements and the transfer curve becomes stable (Fig. S3†). In order to explain the origin of this drift behaviour, we performed reference electrochemistry measurements on unpolished pure 99.9% gold foil. It is apparent from the CVs characteristics employing different scan rates (Fig. S4†) that the reduction and oxidation of the gold species, namely in the 0.4–0.7 V and 0.1–0.3 V ranges respectively, depending on the scan rate, reflect an irreversible regime. This could be an indication of the charge imbalance attributed to gold dissolution, as has been reported for aqueous solutions.^42^ Dissolution has been linked to defects present on the gold surface; namely, in the different ionic states of Au, O/OH adsorption and Au–O/OH place exchange. This process could take a few cycles to saturate in the case of an unpolished/uncycled sample due to the saturation of the surface towards a reversible regime.^42^ Therefore, during the measurements of sensing each measurement was repeated five times before recording the transfer curve. We further determined the measurement stability in PBS by recording the change in transfer curves over time. The *I*–*V* curve shows a stable Dirac voltage from 15 to 120 min (Fig. 3H), indicating good stability of the GFET device in buffer solution. Additionally, we examined the uniformity in device-to-device response for the detection of target spike proteins at 300 pM in d1000 PBS. As seen in Fig. 3I, the transfer curves show minimal device-to-device variation. The Dirac voltage remains at 0.67 V and the relative standard deviations (RSDs) of *V*_D_ are below 0.5%. This indicates that the on-chip GFET biosensors offer very competitive repeatability in the measurement of analytes compared to other reported nanosensors.^43^ The high stability of the on-chip sensor in buffer solution along with its low cost and compatibility with microfabrication processes make it suitable for large-scale development in POC settings.

We then demonstrated the importance of internal control measurement and multiplexed detection for on-chip sensing. The performance of the on-chip GFETs sensors was assessed *via* the simultaneous monitoring of transfer curves measured with multiple devices on one chip, for internal controls (PBS buffer without any target proteins) and for spike protein samples (33 pM). The *I*_SD_–*V*_GS_ curves from 5 GFETs show very minimal change in the Dirac point for the PBS samples (Fig. S5A†), but a noticeable shift to the right for the target samples (Fig. S5B†). Fig. S5C† shows a comparison of signal changes for the internal controls (*n* = 5) and tested target samples (*n* = 5) and a significant difference was observed (*p* < 0.001, ***). The subtraction of the internal control signal as background and averaging over several GFETs represents a viable approach to increase the statistical significance of a test result, in particular with regards to the identification of GEFTs that do not operate properly. We consider this as an important advantage over standard COVID-19 tests such as PCR or lateral flow which are ‘one-channel’ tests.

In summary, internal on-chip control measurements, along with the excellent repeatability and stability allowed during the measurement of liquid samples give this on-chip GFET platform outstanding accuracy and robustness for bioanalysis and bio-detection.

### 3.2 Analytical performance of the on-chip integrated graphene biosensor

**Fig. 4:**
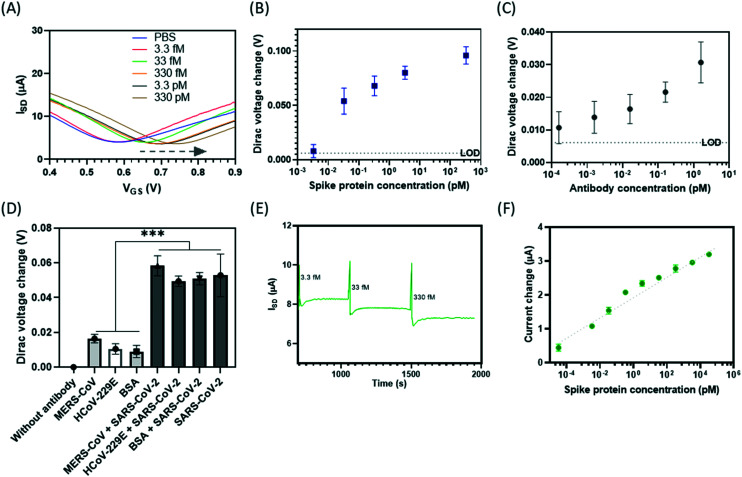
Analytical performance assessment of the on-chip GFET sensing platform for SARS-CoV-2 spike protein detection: (A and B) sensitivity; (A) *I*–*V* curves showing the Dirac point shift with an increasing spike protein concentration from 3.3 fM to 330 pM compared to the PBS (blank) sample; (B) calibration curves of Dirac voltage change against the spike protein concentration in the d1000 PBS buffering condition (*y* = 0.0069 ln(*x*) + 0.0657; *R*^2^ = 0.8433), where the LOD can be as low as 3 fM (10^3^ proteins per μL) which is equal to 10–10^2^ viruses per μL; (C) the serum testing using the on-chip GFET sensor (the detection of various concentrations of antibody dilutions) (*n* = 5); (D) the specificity test of spike protein antibodies against other non-target proteins using GFET sensors, where the bar chart shows the comparison of the change in Dirac point between the samples tested at 165 pM. *n* = 4; (E) the real-time monitoring of the source–drain current (*I*_SD_) change over time (s) in response to a series of spike protein concentrations, where *V*_SD_ = 20 mV, *V*_G_ = 0.65 V and the response time of the GFET sensor can be as fast as 100–150 s; (F) the change in current *vs.* the spike protein concentration.

We assessed the analytical performance of the developed on-chip integrated GFET biosensor (Ab-GFET) for the detection of SARS-CoV-2 by targeting surface antigen spike proteins. Key parameters of the development of GFET biosensor were first optimized, including incubation methods for the linker PBASE and the antibody, the ionic strength of the tested PBS buffer, and antibody concentration (Fig. S6†). The *I*–*V* curves show that the Dirac point shifted to the right with increasing spike protein concentrations from 3.3 fM to 3.3 nM compared to d1000 PBS buffer (blank) sample (Fig. 4A). Fig. 4B shows the calibration curve of the change in Dirac voltage against target concentration (*y* = 0.0069 ln(*x*) + 0.0657; *R*^2^ = 0.8433). As determined by the blank signal (PBS) plus three-times the standard deviations (SD) of the blank control, the limit-of-detection (LOD) is determined to be 3 fM (10^3^ protein molecules per μL), which equals 10–10^2^ viruses per μL if one viral particle contains 100 surface spike proteins.^40^

The ultra-sensitivity of the Ab-GFET biosensor was also demonstrated for the detection of neutralizing antibody using a similar biosensor fabricated with this on-chip platform. We first immobilized the recombinant antigen protein (with the spike protein as an example) by conjugation to the linker molecule PBASE *via* the NHS group (Fig. S8A†). The prepared GFET sensors were then deployed to measure a series of antibody dilutions with concentrations down to aM level in PBS buffer (Fig. S7†). We then prepared antibody stocks in serum so as to mimic a patient's COVID-19-positive blood sample and tested the antibody samples using this on-chip biosensor. As shown in Fig. S8B,† the transfer curve retains its shape and the Dirac voltage shifts to the right with increasing antibody concentration in serum from 160 aM to 1.6 pM. The measured Δ*V*_D_ values are compatible with the expected logarithmic dependence on concentration, with an LOD (defined as blank + 3 SD) of 160 aM (Fig. 4C). This demonstrates the ultra-sensitivity of this on-chip GFET biosensor for bio-detection even with real samples such as serum, thus outperforming the current lateral flow tests which give large percentages of false-negatives.^44,45^ Moreover, the demonstration shows the ability in the rapid screening of antibodies in serum-containing conditions, offering a robust alternative for the testing of those infected and tracing the history of infection with COVID-19.

Besides this, the specificity of the Ab-GFET biosensor was investigated using the spike antibody-coated on-chip GFET sensors, with six devices for the testing pf target proteins and the other six devices for non-targets as internal controls. The *I*–*V* curves show a much larger shift to the right of the Dirac point for the samples containing mixtures of the MERS-CoV spike + SARS-CoV-2 spike proteins (Fig. S9A†), HCoV-229E + SARS-CoV-2 spike proteins (Fig. S9B†), and BSA + SARS-CoV-2 spike proteins (Fig. S9C†) compared respectively with samples of control MERS-CoV spike protein only, HCoV-229E spike protein only or BSA only. The bar chart in Fig. 4D compares changes in the values of the Dirac point between these samples tested at 165 pM. We also monitored real-time changes in the source–drain current (*I*_SD_) over time (in units of seconds) in response to a series of concentrations of spike protein samples at *V*_SD_ = 20 mV and *V*_G_ = 0.65 V, as shown in Fig. 4E and S10.† The GFET sensor responds to the introduction of samples in real-time and the response time can be as fast as 100–150 s (within 3 minutes). As with the Dirac point shift, the change in current is logarithmic depending upon spike protein concentration,^46^ as plotted in Fig. 4F, indicating an LOD below the concentration of 3 fM. This agrees with the results obtained by measuring the shift in Dirac voltage (Fig. 4B). This ultra-sensitivity proves the excellent performance of the on-chip GFET sensors.

### 3.3 Apt-GFET outperforms Ab-GFET in detecting SARS-CoV-2 viruses (VLPs) in UTM

Although the antibody-functionalized GFETs (Ab-GFET) exhibit versatile detection capability for proteins and antibodies as targets, the large size (10 nm) of an antibody can limit the sensor's response due to the screening effect. In recent years, aptamers which are single-stranded nucleic acid sequences with high affinity for biomolecules have been proposed as alternatives to antibodies in the design of biosensors.^47–51^ Due to their generally smaller size compared to antibodies and greater flexibility in providing specific binding conformation, aptamers are hypothesized to enhance the sensitivity of the proposed GFETs.

**Fig. 5:**
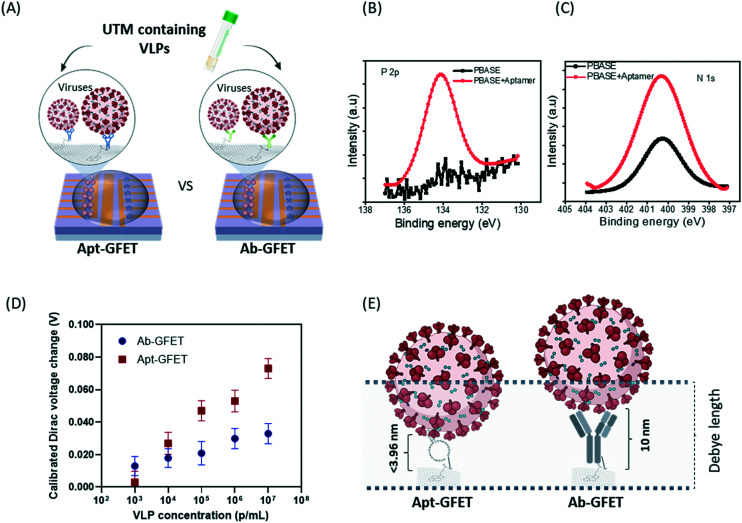
The SARS-CoV-2 detection in universal transport media (UTM) (mimic swab sample) using the on-chip integrated graphene biosensor (Apt-GFET, aptasensor): (A) the comparison schematic of the on-chip Apt-GFET biosensor (aptasensor) *vs.* the Ab-GFET one; (B) P 2p XPS spectra; and (C) N 1s XPS spectra confirm the functionalization/attachment of aptamers on the graphene surface; (D) results of both sensors for SARS-CoV-2 virus detection in UTM; (E) the scheme showing the Debye screen effect for the Apt-GFET and Ab-GFET, as well as a comparison of the sizes of the antibody and aptamer.

In this study, the Apt-GFET biosensor was used for the detection of SARS-CoV-2 viruses (VLPs) in real universal transport medium (UTM) in comparison to the Ab-GFET counterpart (Fig. 5A). We first surface-conjugated the on-chip GFETs with NH_2_-aptamer *via* the linker PBASE, which was confirmed using XPS measurement (Fig. 5B and C). To mimic swab samples from COVID-19-positive patients, the virus-like particle (VLP) stock sample (10^9^ particles per mL) was mixed with UTM. The VLP stock sample was then diluted to concentrations of 10^3^–10^7^ particles per mL using d1000 PBS and detected on the GFET chip. The GFET sensor is so sensitive that anything apart from the target may interfere with the detection signal. Therefore, utilizing the internal control-enabled on-chip GFETs, the interference electrical signals caused by the supplier's buffer and UTM at the same dilution level were also measured on the same chip simultaneously. The change in Dirac voltage caused by the existence of VLP was calibrated by subtracting the buffer/UTM-only signal for each corresponding sample and the results are plotted in Fig. 5D. The graph shows that the on-chip Apt-GFET sensors show significant response at VLP concentrations from 10^3^–10^7^ particles per mL, where the LOD of on-chip GFETs for whole virus detection could be 10–10^2^ viruses per μL or even lower (single particle detection). The buffer/UTM-only sample shows a net zero Dirac voltage shift (baseline) after normalization, which demonstrates the self-correcting and error-reducing characteristics of this sensor. The response of Apt-GFET was stronger than that of Ab-GFET, again confirming the enhancement of GFET sensitivity by using the aptamer. The chosen buffer condition gives a Debye screening length of approximately 23 nm.^12^ Due to the shorter length of aptamer compared to that of the antibody (Fig. 5E), the majority of viral particle charges are unscreened by counter-ions in the solution. This consequently leads to a greater contribution to the sensor response and to the higher detection sensitivity. As is known, the IgG antibodies (∼150 kDa) are about 10–15 nm in size^52^ and arranged in three globular regions that roughly form a Y shape, whereas the aptamer chain used in this study is 22 to 26 Å in in width (2.2–2.6 nanometers), and one nucleotide unit was measured as 3.3 Å (0.33 nm) long. Hence the width and length of the folded aptamer structure could be shorter than 3.96 nm, representing a halving of dimensions compared to the antibody. Reorientation occurred upon the binding of aptamers, and therefore we believe that both the target and a substantial portion of the negatively-charged backbones of the aptamer^48^ have moved closer to the graphene surface where screening is weaker, and therefore the strength of the signal is dramatically increased. In other words, a larger amount of charge is located within the range of the Debye length, therefore largely facilitating the extreme sensitivity of our GFET sensors.

A summary comparison with more recent work shown in Table S1† suggests that our devices represent one of the most promising types of GFET biosensor with unpreceded sensitivity for biomedical applications in disease detection. Aptamers can be developed against almost every target and chemically synthesized with large output at low cost, and they are stable at room temperature and have long shelf-lives.^53^ These advantages allow Apt-GFETs to outperform other sensors, which could lead to a considerable extension of their application for more robust bio-detection.

Our results demonstrate that the proposed on-chip integrated Apt-GFET (aptasensor) platform comprising real-time, label-free, and fast biosensing capability in the order of minutes is a sensitive and specific detection tool for the analysis of SARS-CoV-2 whole viral particles in real medium.

## 4 Conclusions

In this work, a compact, highly reproducible, and in-plane GFET platform as a label-free, ultrasensitive, and selective biosensing tool has been demonstrated. We showed that an on-chip Au electrode is suitable as a liquid gate electrode for GFET sensor operation that can simplify the implementation of GFETs in biosensing applications. Using this on-chip GFET platform, high performance COVID-19 detection with the antibody functionalization was achieved. The electrical detection of GFET was further improved using aptamer functionalized graphene surface. The aptamer-facilitated GFET platform (Apt-GFET) for SARS-CoV-2 detection exhibits ultra-sensitivity with the limits of detection down to 10^3^ particles per mL for 100 nm-ranged viral particles in virus transport medium and 160 aM for COVID-19 neutralizing antibodies in serum. The developed GFETs will be evaluated for testing virus infection in clinical samples and compared with gold standard methods in the future. We believe that this sensor platform with portable electronics could accelerate the transformation of such technology from research to real life.

## Conflicts of interest

There are no conflicts to declare.

## Supplementary Material

SD-001-D2SD00076H-s001
