# Supplementary material for: On-chip integrated graphene aptasensor with portable readout for fast and label-free COVID-19 detection in virus transport medium
Source: Sens Diagn. 2022 Jun 13;1(4):719–30. doi: 10.1039/d2sd00076h (PMC9280445; doi:10.1039/d2sd00076h)
Supplement: SD-001-D2SD00076H-s001 [file SD-001-D2SD00076H-s001.pdf]

Electronic Supplementary Material (ESI) for Sensors & Diagnostics.
This journal is © The Royal Society of Chemistry 2022

Supporting information for

On-chip integrated graphene aptasensor with portable readout for fast and label-free COVID-19 detection in virus transport medium

Lizhou Xu[1, 2, #, *], Sami Ramadan[1, #], Bruno Gil Rosa[3], Yuanzhou Zhang[1], Tianyi Yin[1], Elias Torres[4], Olena Shaforost[1], Apostolos Panagiotopoulos[1], Bing Li[5, 6, 7], Gwilherm Kerherve[1], Dong kuk Kim[1], Cecilia Mattevi[1], Long R. Jiao[8], Peter K. Petrov[1], and Norbert Klein [1, *]

[1] Department of Materials, Imperial College London, London, SW7 2AZ, UK

[2] ZJU-Hangzhou Global Scientific and Technological Innovation Center, Zhejiang University Hangzhou, 311215, China

[3] Hamlyn Centre, Imperial College London, London, SW7 2AZ, UK

[4] Graphenea Semiconductor, Paseo Mikeletegi 83, San Sebastián, 20009, Spain

[5] Department of Brain Sciences, Imperial College London, London, W12 0BZ, UK

[6] Care Research & Technology Centre, UK Dementia Research Institute, W12 0BZ, UK

[7] Institute for Materials Discovery, University College London, Roberts Building, London, WC1E 7JE, UK

[8] Department of Hepatobiliary Surgery, Division of Surgery & Cancer, Imperial College London, Hammersmith Hospital Campus, Du Cane Road, London, W12 0NN, UK

# These authors contributed equally.

* Corresponding authors:

Dr. Lizhou Xu, l.xu@imperial.ac.uk

Prof. Dr. Norbert Klein, n.klein@imperial.ac.uk

Raman Spectroscopy

Raman spectroscopy measurements were performed using a confocal Witec spectrometer and excited with laser wavelength of 532 nm (excitation energy $E_L = \hbar w_L = 2.33\ eV$) through an optical fibre, and an objective lens of 100X, NA=0.8 and laser spot of 0.4 µm. The laser power was kept below 2 mW and spectral resolution was ~3 cm^{-1}; the Raman peak position was calibrated based on the Si peak position at 520.7 cm^{-1}. The D, G and 2D peaks were fitted with Lorenzian functions.

X-ray Photoelectron Spectroscopy (XPS)

XPS experiments and measurements were performed with K-Alpha+ and an Al radiation source ($hv = 1486.6\ eV$) in an ultrahigh vacuum chamber for spectroscopic analysis with a base pressure of 5×10^{-8} mbar.

Atomic Force Microscopy (AFM) Measurements

AFM was performed using Asylum MFP-3D classic and a Bruker Innova system in tapping mode with commercial tips of average radius 15 nm and typical scan resolution of 512 pixel X 512 pixel. All AFM scans were performed in dry conditions.

Electrochemical Measurements

A number of cyclic voltammograms were performed in the 10-1000 mV/s scan rate range, reflecting the sensing operating conditions of 1-100 seconds. Each scan rate was cycled 10 times and the third of each was plotted. Ag/AgCl reference electrode was purchased by BioAnalytical Systems Inc. (BASi) and stored in a 3 M KCl solution before usage. Ag/AgCl Reference electrode was calibrated with an identical Master Reference Electrode that is non-cycled and stored in controlled conditions of 3 M KCl solution at 25°C and remeasured after any series of electrochemical measurements, without conditioning. Working electrode contacted with a flat-end crocodile clip that was clamped above the electrolyte surface. The same amount of the device's surface area was immersed into the 0.001 M PBS electrolyte, at a pH level of 7.4 and 40 mL in volume, for each experiment. Counter electrode was 1 cm^2 of Carbon foil purchased from Merck. All electrodes were rinsed thoroughly with DI and air-dried before cell assembly.

Surface charge measurement

The surface charge (zeta potential) of purified exosomes and labelled exosomes in PBS were measured using a Zetasizer Nano Series ZS with illumination from a 633 nm He-Ne laser (Malvern Instruments, Malvern, UK). Samples were diluted into approximately 10^8 particles/mL concentration using 10 times diluted PBS and then transferred to a disposable plain folded capillary Zeta cell. Zeta potential was determined from the electrophoretic mobility using the Smoluchowski approximation. Measurements were carried out at room temperature.

Nanoparticle Tracking Analysis

The size distribution and particle number/concentration of exosomes were measured by nanoparticle tracking analysis (NTA) using a Nanosight LM10 system with blue (488 nm) laser (Malvern Instruments, Worcestershire, UK). Exosomes were diluted in filtered deionised water to obtain 20~60 vesicles per field of view for optimal tracking. Three videos of 30 s were taken and analysed using the NanoSight NTA 3.2 software. The area under the histogram for each triplicate measurement was averaged and used as one particle concentration measurement. All NTA measurements were done with identical system settings for consistency.

Antibody Testing

The samples were first prepared in d1000 PBS as a proof-of-concept study. In order to mimic the COVID-19 antibody positive patient sample, the antibody stock (3.3 µM) were mixed with commercial supplied serum at 1:1 ratio (v:v). The sample was then diluted to a serial antibody concentration by d1000 PBS until 160 aM before testing using this on-chip biosensor. For testing the neutralizing antibodies for COVID-19, each chip was incubated with SARS-CoV-2 (2019-nCoV) spike S1 recombinant protein (40591-V08H, Sino Biological, Germany).

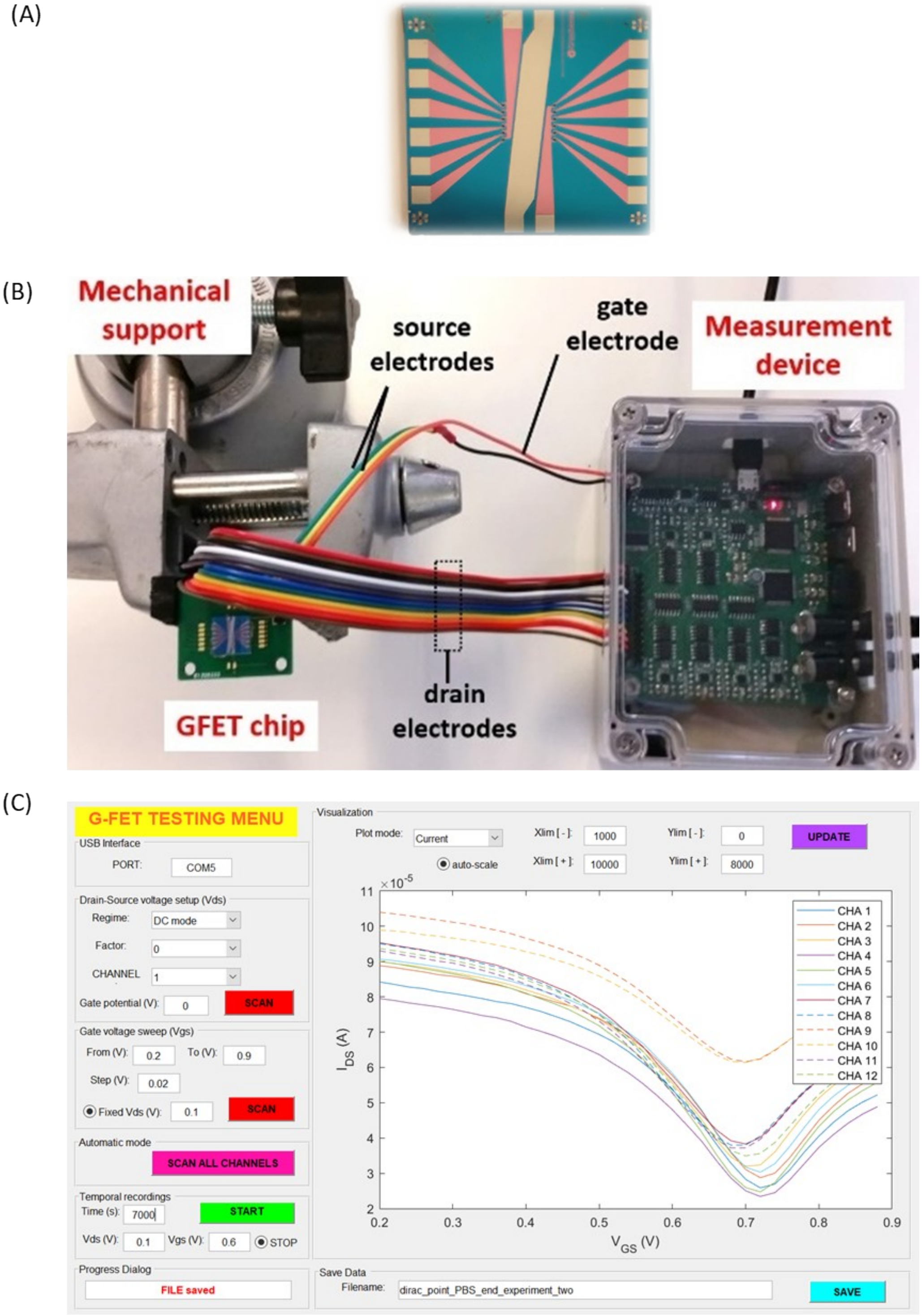

Figure S1. Portable measurement device involved in GFET measurements. (A) Image of the on-chip GFET sensor array. (B) Measurement setup with the proposed device connected to the GFET chip. (C) Graphical user interface developed to control the device and visualize the acquired signals from the GFETs on the computer side.

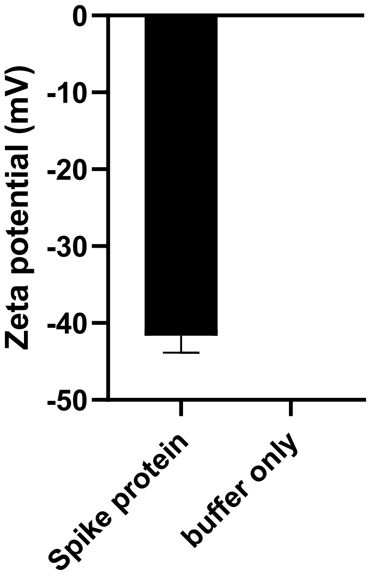

Figure S2. The zeta potential of SARS-CoV-2 spike protein (30 pM) in testing buffer d1000 PBS.

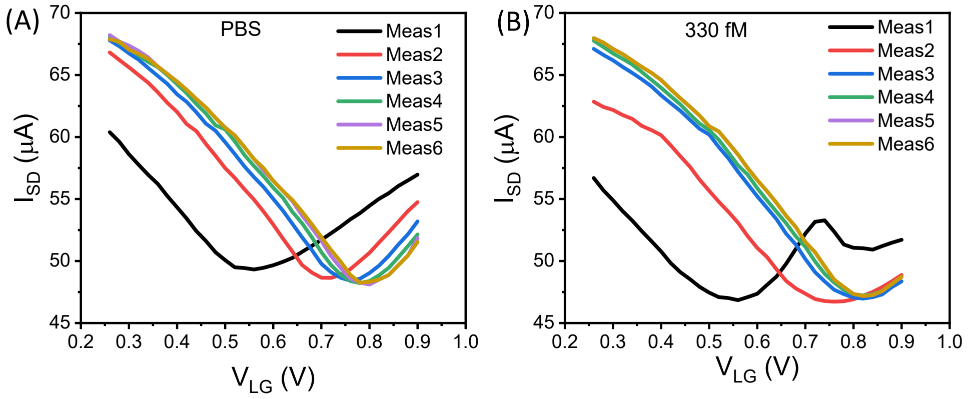

Figure S3. Series of repeated transfer curve measurements of GFETs (A) in PBS; (B) after injection of spike protein (330 fM). The data shows that the transfer curve become stable after the 5th measurements in both cases. Injection of 330 fM causes a shift of 40 mV in Dirac voltage compared to PBS.

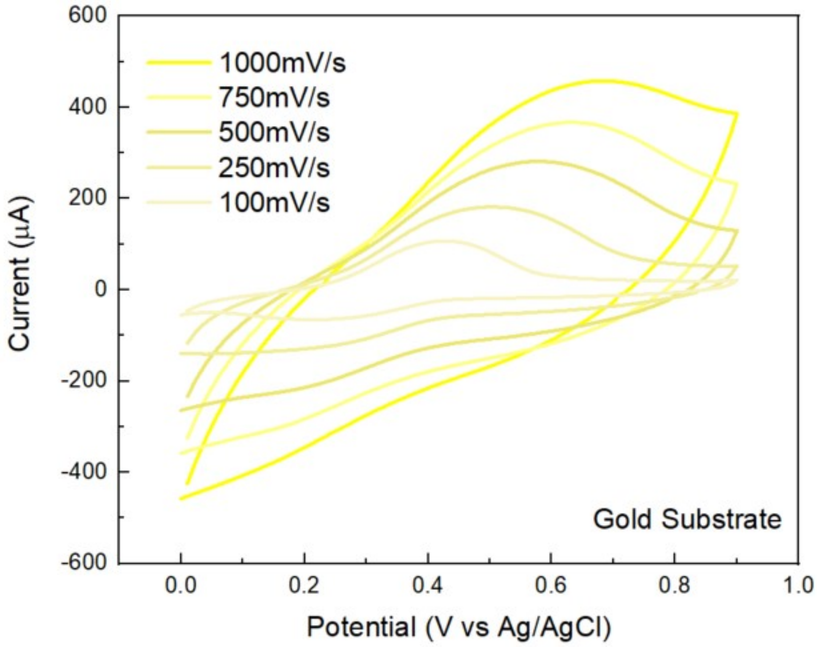

Figure S4. Electrochemistry of the pure gold in a three-electrode setup with an Ag/AgCl reference electrode and carbon foil counter electrode in 0.001M PBS solution of pH 7.4.

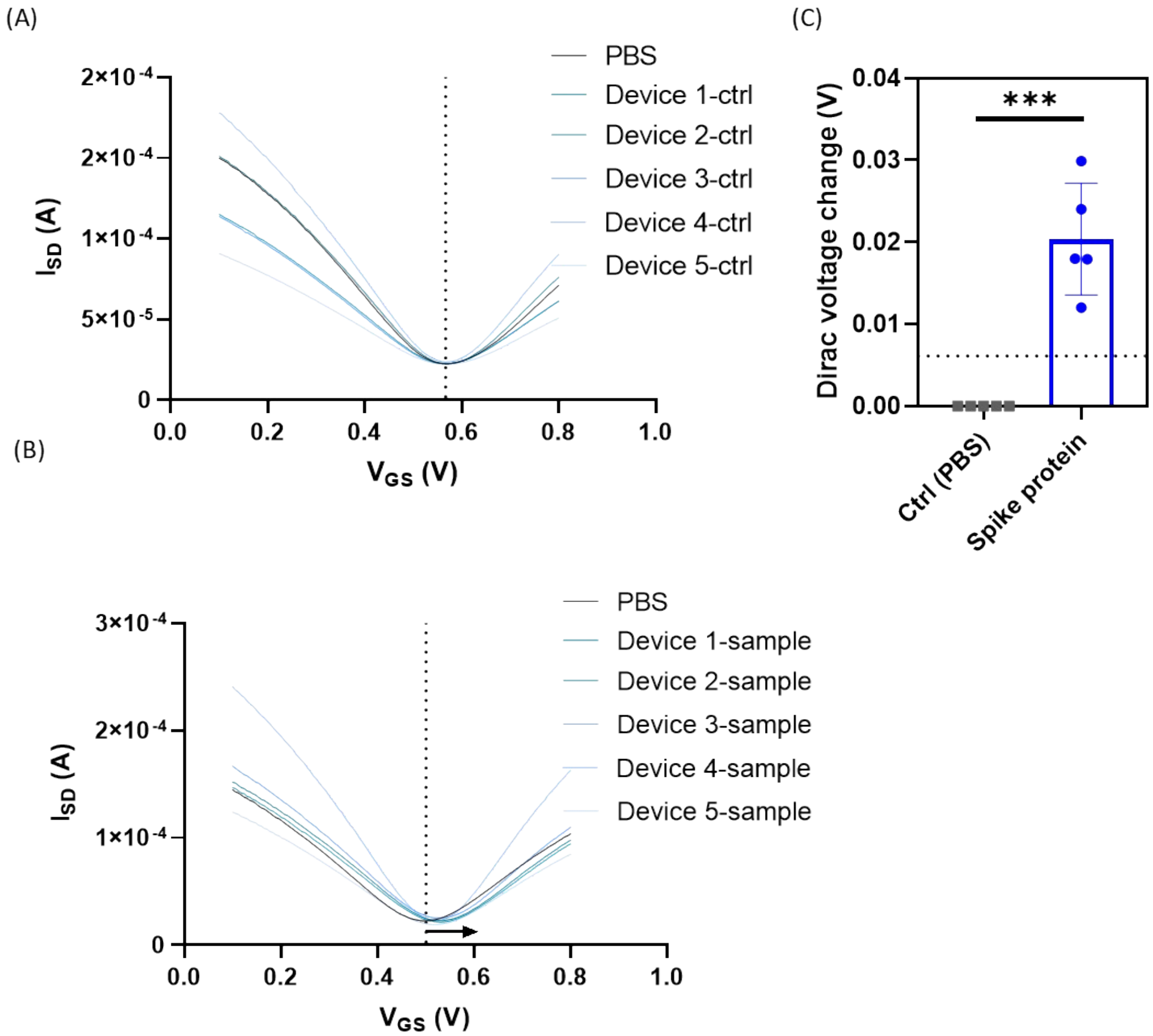

Figure S5. On-chip internal control and performance assessment of GFET sensors: (A-C) signal monitoring of multiple devices for internal control (PBS buffer without any target proteins) and SARS-CoV-2 spike protein samples (33 pM). I-V curves from 5 devices showing no change of Dirac point in PBS samples (A), whereas a significant shift to the right is shown for target samples (B); (C) comparison of signal changes for the internal controls and tested target samples, n=5. $P<0.001$,***.

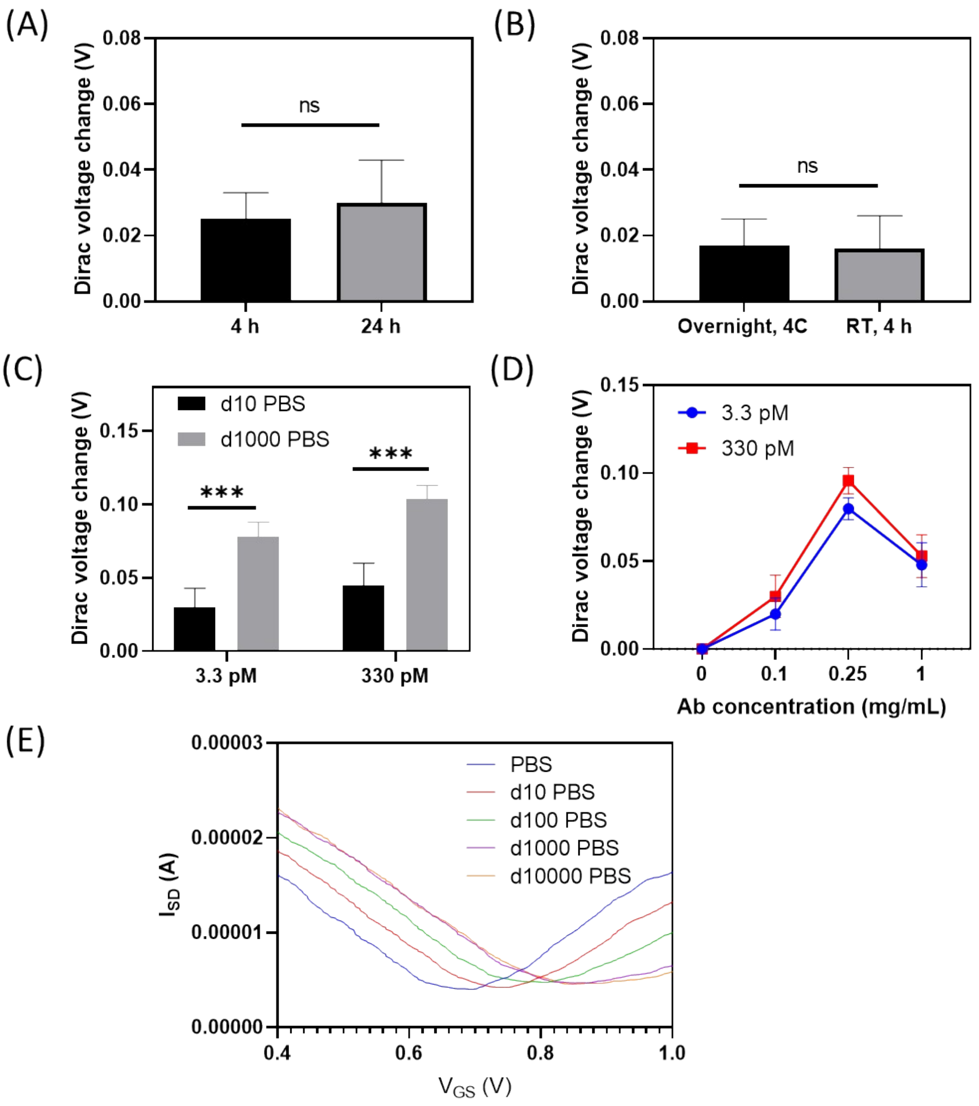

Figure S6. Optimization of some key conditions of the development of GFET sensors. (A) The linker molecule PBASE incubation methods. (B) The antibody incubation methods. (C) Comparison between buffers with different ionic strengths. (D) Antibody concentration. (E) Ionic strength of different PBS detection solution.

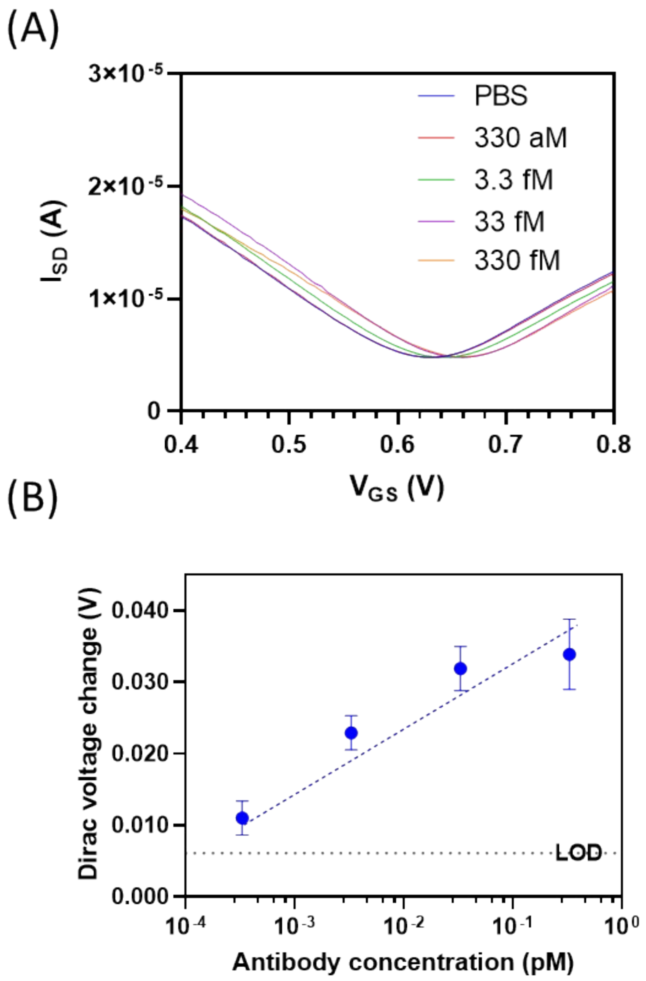

Figure S7. Antibody testing in PBS buffer by the on-chip GFET sensors for COVID-19. (A) Transfer characteristics and (B) Calibration curves of the GFETs in detecting a range of concentrations of antibody dilutions.

(A)

(B)

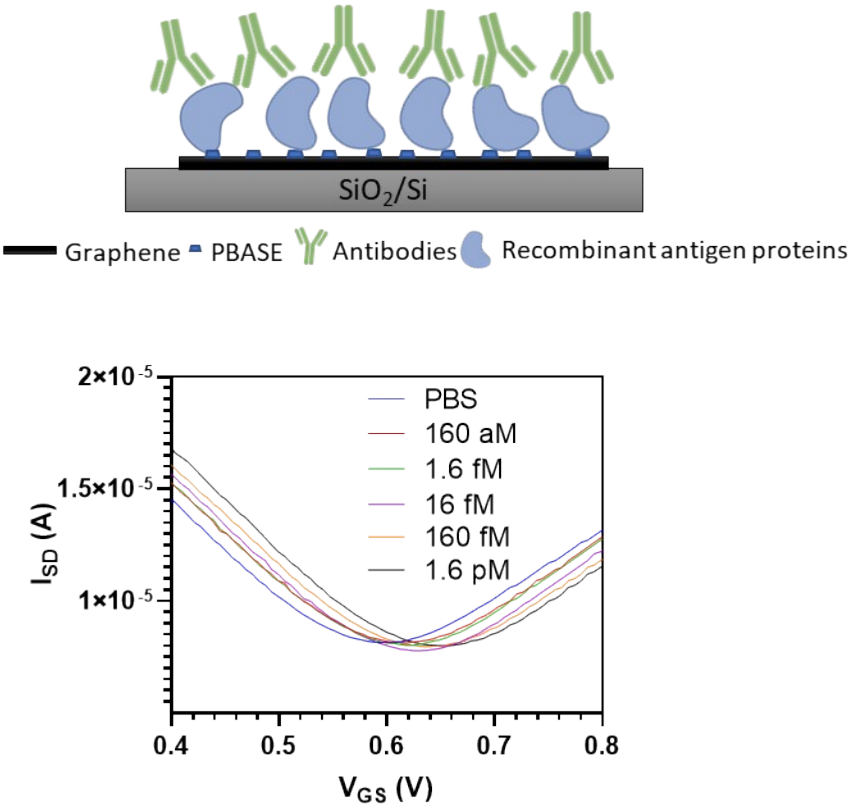

Figure S8. Antibody testing in serum by the on-chip GFET sensors for COVID-19. (A) Scheme of the GFET design using recombinant spike protein antigens immobilized onto the GFET surface; (B) Transfer characteristics of the GFET sensor for the detection of a range of antibody dilutions in serum (n=5).

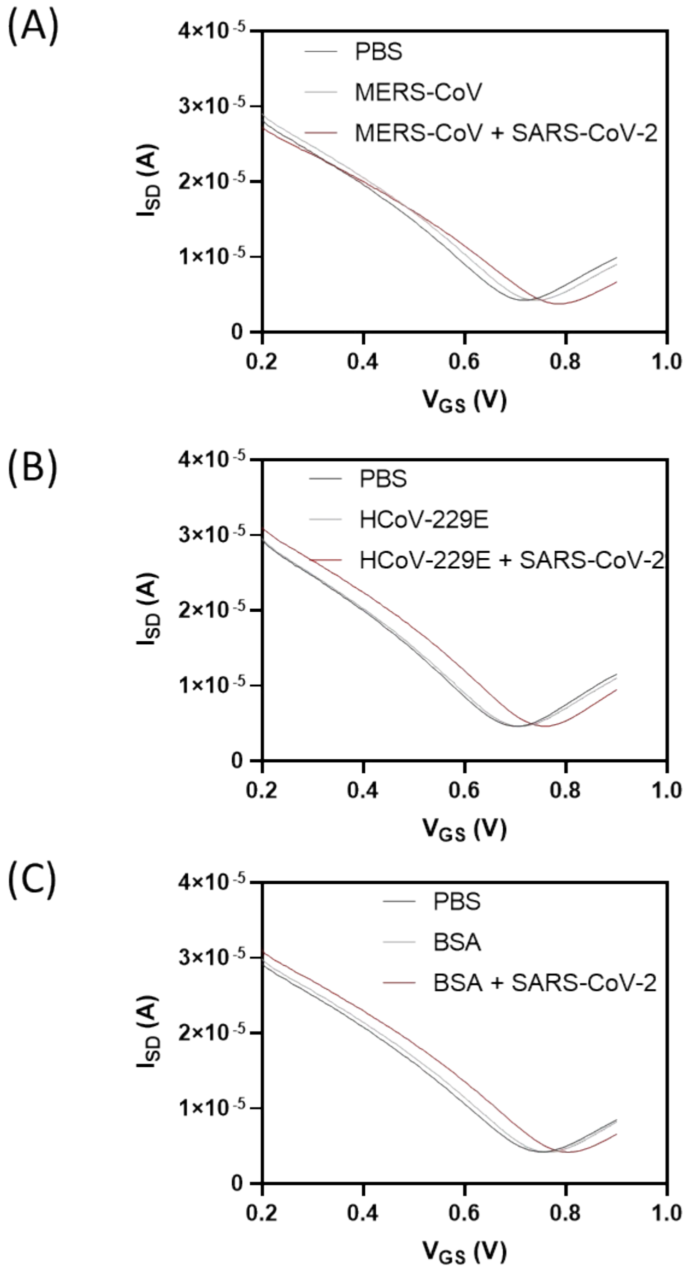

Figure S9. Specificity test of spike protein antibody against other non-target proteins using GFET sensors. I-V curves showing much larger right shift of Dirac point for (A) MERS-CoV spike protein + SARS-CoV-2 spike protein mixture samples, (B) HCoV-229E + SARS-CoV-2 spike protein mixture, and (C) BSA + SARS-CoV-2 spike protein mixture samples than the control MERS-CoV spike protein only, HCoV-229E spike protein only or BSA only sample, respectively.

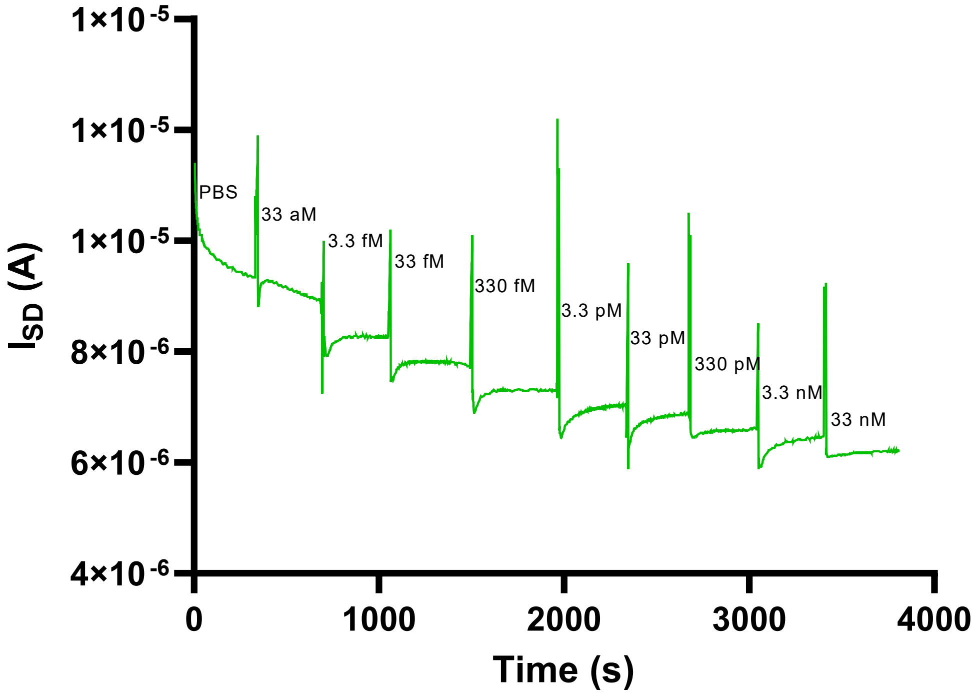

Figure S10. Real time monitoring of the source-drain current (I_{SD}) change by time (s) in response to a series of spike protein concentrations. V_{SD}=20 mV, V_G=0.65 V. Response time of the GFET sensor can be as fast as 100-150 s.

Table S1. Comparison of key parameters of the GFET biosensors reported recently for diseases detection from 2015.

**Target**	**Molecular weight (kD)**	**Biorecognition element**	**GFET type[1]**	**LOD**	**Ref.**
Spike protein	76.5	Ab	On-chip devices	3 fM	This work
IgG antibody	150	Recombinant antigen protein	On-chip devices	330 aM	This work
VLPs	>>150	Ab	On-chip devices	10^5 p/mL	This work
VLPs	>>150	Apt	On-chip devices	10^3 p/mL	This work
Exosomes	>>150	Ab	Conventional	10^5 p/mL	1
Exosomes	>>150	Ab	Conventional (back gate)	0.1 µg/mL (~10^7 p/mL)	2
Geosmin	0.18	Apt	Conventional	10 pM	3
Cu^{2+}	0.06	CQDs	Conventional	10 fM	4
DNA	~2	DNA	Deformed conventional GFET	20 aM	5
DNA	~2	Apt	Conventional + AuNPs	15 aM	6
Lactose	0.34	Protein	Conventional + AuNPs	200 aM	7
Neuropeptide Y (NPY)	4.3	Peptide	Conventional	1 pM	8
Spike protein	76.5	Ab	Conventional	1 fg/mL (13 aM)	9
Interleukin-6 (IL-6)	21	Apt	Conventional	618 fM	10
Insulin	5.8	Apt	Conventional	766 fM	10
Biotin	0.24	Avidin	Conventional (six devices)	370 fM	11
17β-estradiol	0.27	Apt	Conventional	34.7 pM	12
Penicillin	0.33	Enzyme	Conventional+ hydrogel	0.2 mM	13
Gene	~6	dCas9-sgRNA	Conventional+ CRISPR/Cas9	1.7 fM	14
DNA	<5	DNA	Conventional	1 fM	15
Bisphenol A	0.23	DNA	Conventional+ microfluidics	10 ng/mL (~4 nM)	16
Brain natriuretic peptide (BNP)	3.5	Ab	Conventional + PtNPs	100 fM	17
DNA	3.3	Peptide nucleic acid (PNA)	Conventional	4 pM	18
PSA	26	Ab	Conventional (rGO-FET)	1.7 fM	19
miRNA	~6	Peptide nucleic acid (PNA)	Conventional+ AuNPs	10 fM	20

Note: 1. Unless stated separately, all the examples listed here are top/electrolyte gated GFET sensors.

Reference

(1) Ramadan, S.; Lobo, R.; Zhang, Y.; Xu, L.; Shaforost, O.; Kwong, D.; Tsang, H.; Feng, J.; Yin, T.; Qiao, M.; et al. Carbon-Dot-Enhanced Graphene Field-Effect Transistors for Ultrasensitive Detection of Exosomes. *ACS Appl. Mater. Interfaces* **2021**. https://doi.org/10.1021/acsami.0c18293.

(2) Kwong Hong Tsang, D.; Lieberthal, T. J.; Watts, C.; Dunlop, I. E.; Ramadan, S.; del Rio Hernandez, A. E.; Klein, N. Chemically Functionalised Graphene FET Biosensor for the Label-Free Sensing of Exosomes. *Sci. Rep.* **2019**, *9* (1). https://doi.org/10.1038/s41598-019-50412-9.

(3) Park, S. J.; Seo, S. E.; Kim, K. H.; Lee, S. H.; Kim, J.; Ha, S.; Song, H. S.; Lee, S. H.; Kwon, O. S. Real-Time Monitoring of Geosmin Based on an Aptamer-Conjugated Graphene Field-Effect Transistor. *Biosens. Bioelectron.* **2021**, *174*, 112804. https://doi.org/10.1016/j.bios.2020.112804.

(4) Fan, Q.; Li, J.; Zhu, Y.; Yang, Z.; Shen, T.; Guo, Y.; Wang, L.; Mei, T.; Wang, J.; Wang, X. Functional Carbon Quantum Dots for Highly Sensitive Graphene Transistors for Cu2+ Ion Detection. *ACS Appl. Mater. Interfaces* **2020**, *12* (4), 4797–4803. https://doi.org/10.1021/acsami.9b20785.

(5) Hwang, M. T.; Heiranian, M.; Kim, Y.; You, S.; Leem, J.; Taqieddin, A.; Faramarzi, V.; Jing, Y.; Park, I.; van der Zande, A. M.; et al. Ultrasensitive Detection of Nucleic Acids Using Deformed Graphene Channel Field Effect Biosensors. *Nat. Commun.* **2020**, *11* (1), 1543. https://doi.org/10.1038/s41467-020-15330-9.

(6) Danielson, E.; Sontakke, V. A.; Porkovich, A. J.; Wang, Z.; Kumar, P.; Ziadi, Z.; Yokobayashi, Y.; Sowwan, M. Graphene Based Field-Effect Transistor Biosensors Functionalized Using Gas-Phase Synthesized Gold Nanoparticles. *Sensors Actuators, B Chem.* **2020**, *320*, 128432. https://doi.org/10.1016/j.snb.2020.128432.

(7) Danielson, E.; Dindo, M.; Porkovich, A. J.; Kumar, P.; Wang, Z.; Jain, P.; Mete, T.; Ziadi, Z.; Kikkeri, R.; Laurino, P.; et al. Non-Enzymatic and Highly Sensitive Lactose Detection Utilizing Graphene Field-Effect Transistors. *Biosens. Bioelectron.* **2020**, *165*. https://doi.org/10.1016/j.bios.2020.112419.

(8) Islam, A. E.; Crasto, C. M.; Crasto, C. M.; Kim, S. S.; Rao, R. S.; Maruyama, B.; Drummy, L. F. Graphene-Based Electrolyte-Gated Field-Effect Transistors for Potentiometrically Sensing Neuropeptide y in Physiologically Relevant Environments. *ACS Appl. Nano Mater.* **2020**, *3* (6), 5088–5097. https://doi.org/10.1021/acsanm.0c00353.

(9) Seo, G.; Lee, G.; Kim, M. J.; Baek, S. H.; Choi, M.; Ku, K. B.; Lee, C. S.; Jun, S.; Park, D.; Kim, H. G.; et al. Rapid Detection of COVID-19 Causative Virus (SARS-CoV-2) in Human Nasopharyngeal Swab Specimens Using Field-Effect Transistor-Based Biosensor. *ACS Nano* **2020**, *14* (4), 5135–5142. https://doi.org/10.1021/acsnano.0c02823.

(10) Hao, Z.; Pan, Y.; Huang, C.; Wang, Z.; Lin, Q.; Zhao, X.; Liu, S. Modulating the Linker Immobilization Density on Aptameric Graphene Field Effect Transistors Using an Electric Field. *ACS Sensors* **2020**, *5* (8), 2503–2513. https://doi.org/10.1021/acssensors.0c00752.

(11) Wang, S.; Hossain, M. Z.; Shinozuka, K.; Shimizu, N.; Kitada, S.; Suzuki, T.; Ichige, R.; Kuwana, A.; Kobayashi, H. Graphene Field-Effect Transistor Biosensor for Detection of Biotin with Ultrahigh Sensitivity and Specificity. *Biosens. Bioelectron.* **2020**, *165*, 112363. https://doi.org/10.1016/j.bios.2020.112363.

(12) Li, Y.; Zhu, Y.; Wang, C.; He, M.; Lin, Q. Selective Detection of Water Pollutants Using a Differential Aptamer-Based Graphene Biosensor. *Biosens. Bioelectron.* **2019**, *126*, 59–67. https://doi.org/10.1016/j.bios.2018.10.047.

(13) Bay, H. H.; Vo, R.; Dai, X.; Hsu, H. H.; Mo, Z.; Cao, S.; Li, W.; Omenetto, F. G.; Jiang, X. Hydrogel Gate Graphene Field-Effect Transistors as Multiplexed Biosensors. *Nano Lett.* **2019**, *19* (4), 2620–2626. https://doi.org/10.1021/acs.nanolett.9b00431.

(14) Hajian, R.; Balderston, S.; Tran, T.; deBoer, T.; Etienne, J.; Sandhu, M.; Wauford, N. A.; Chung, J. Y.; Nokes, J.; Athaiya, M.; et al. Detection of Unamplified Target Genes via CRISPR–Cas9 Immobilized on a Graphene Field-Effect Transistor. *Nat. Biomed. Eng.* **2019**, *3* (6), 427–437. https://doi.org/10.1038/s41551-019-0371-x.

(15) Li, S.; Huang, K.; Fan, Q.; Yang, S.; Shen, T.; Mei, T.; Wang, J.; Wang, X.; Chang, G.; Li, J. Highly Sensitive Solution-Gated Graphene Transistors for Label-Free DNA Detection. *Biosens. Bioelectron.* **2019**, *136*, 91–96. https://doi.org/10.1016/j.bios.2019.04.034.

(16) Liu, S.; Fu, Y.; Xiong, C.; Liu, Z.; Zheng, L.; Yan, F. Detection of Bisphenol A Using DNA-Functionalized Graphene Field Effect Transistors Integrated in Microfluidic Systems. *ACS Appl. Mater. Interfaces* **2018**, *10* (28), 23522–23528. https://doi.org/10.1021/acsami.8b04260.

(17) Lei, Y. M.; Xiao, M. M.; Li, Y. T.; Xu, L.; Zhang, H.; Zhang, Z. Y.; Zhang, G. J. Detection of Heart Failure-Related Biomarker in Whole Blood with Graphene Field Effect Transistor Biosensor. *Biosens. Bioelectron.* **2017**, *91*, 1–7. https://doi.org/10.1016/j.bios.2016.12.018.

(18) Fu, W.; Feng, L.; Mayer, D.; Panaitov, G.; Kireev, D.; Offenhäusser, A.; Krause, H. J. Electrolyte-Gated Graphene Ambipolar Frequency Multipliers for Biochemical Sensing. *Nano Lett.* **2016**, *16* (4), 2295–2300. https://doi.org/10.1021/acs.nanolett.5b04729.

(19) Wang, L.; Jackman, J. A.; Ng, W. B.; Cho, N. J. Flexible, Graphene-Coated Biocomposite for Highly Sensitive, Real-Time Molecular Detection. *Adv. Funct. Mater.* **2016**, *26* (47), 8623–8630. https://doi.org/10.1002/adfm.201603550.

(20) Cai, B.; Huang, L.; Zhang, H.; Sun, Z.; Zhang, Z.; Zhang, G. J. Gold Nanoparticles-Decorated Graphene Field-Effect Transistor Biosensor for Femtomolar MicroRNA Detection. *Biosens. Bioelectron.* **2015**, *74*, 329–334. https://doi.org/10.1016/j.bios.2015.06.068.
